# Low-Power Magnetron Sputtering Deposition of Antimonene Nanofilms for Water Splitting Reaction

**DOI:** 10.3390/mi13030489

**Published:** 2022-03-21

**Authors:** Xingli Wang, Junyu Ge, Nicole Ru-Xuan Ang, Kun Liang, Chong-Wei Tan, Hong Li, Beng Kang Tay

**Affiliations:** 1 UMI 3288 CINTRA (CNRS International-NTU-THALES Research Alliances), Nanyang Technological University, Singapore 637553, Singapore; wangxingli@ntu.edu.sg (X.W.); junyu.ge@ntu.edu.sg (J.G.); liangkun@ntu.edu.sg (K.L.); chongwei@ntu.edu.sg (C.-W.T.); ehongli@ntu.edu.sg (H.L.); 2 Centre for Micro- and Nano-Electronics (CMNE), School of Electrical and Electronic Engineering, Nanyang Technological University, Singapore 638798, Singapore; rang011@e.ntu.edu.sg; 3 School of Mechanical and Aerospace, Nanyang Technological University, Singapore 639798, Singapore

**Keywords:** antimonene, low-power magnetron sputtering deposition, hydrogen evolution reaction, oxygen evolution reaction, water splitting reaction

## Abstract

Antimonene (Sb) is a novel kind of two-dimensional (2D) material that is predicted to be promising for various applications, such as water splitting and semiconductor devices. Several methods have been reported to prepare Sb nanoflakes/nanofilms; however, it is still relatively difficult to prepare Sb nanofilms. In this work, a method of low-power magnetron sputtering deposition was used for the preparation of Sb nanofilms with lateral dimensions on the centimeter scale and controllable film thickness. It was found that the control of the deposition temperature is important for the final crystalline structure of the nanofilms. Furthermore, the application of the nanofilms as a catalyst for water splitting (hydrogen evolution reaction (HER) and oxygen evolution reaction (OER)) was demonstrated.

## 1. Introduction

Since the discovery of graphene in 2004 [1,2], various types of two-dimensional (2D) materials have been explored, ranging from insulators (e.g., hexagonal boron nitride) [3], semiconductors (e.g., transition metal chalcogenides and black phosphorous) [4,5,6], to metals and superconductors, and they have shown high potential when used in logic devices [6,7], sensors and detectors [8], batteries, and supercapacitors [9]. They can also work as catalysts for water splitting reactions or CO_2_ reduction [10,11]. Although many kinds of 2D materials have been reported, novel 2D materials are still emerging, among which antimonene shows high potential as a 2D semiconductor [12,13,14].

Antimonene consists of a layer of antimony atoms that form two kinds of allotropes [12,13]. One is the α phase, in which the atoms form a puckered structure, similar to the structure of black phosphorous. The other one is the β phase, in which the Sb atoms connect into a buckled hexagonal structure. Dynamically, both phases are stable, and cohesive energy calculation shows that these two phases can crossover [12,13,14]. Usually, β-Sb is more stable, especially in thick layers. This explains why the natural antimony crystals are β phase ones [15]. In this work, unless otherwise specified, the antimonene refers to the β phase.

Antimonene has demonstrated vast potential for numerous applications. Zhang et al. predicted that the band gap of monolayer antimonene is 2.28 eV, which is suitable for use in transistors with a high on/off ratio [13]. A high mobility of several tens cm^2^V^−1^s^−1^ makes it a good candidate for nanotransistors with an atomically thin channel [12]. Ab initio quantum transport simulation predicted that an antimonene-based transistor with a sub-5 nm channel length could achieve an on-state current as high as 452 µA/µm, implying a fast switching speed [16]. Gu et al. demonstrated a high volumetric capacity of 1226 mAh/cm^−3^, a good cycle performance, and a high-rate capability of antimonene nanosheets used for sodium storage [9,17]. Antimonene has also been used as near-infrared optothermal agents and demonstrated notable tumor ablation ability in effective cancer therapy [18]. Moreover, antimonene shows potential as a promising catalyst for water splitting reactions. Ren et al. demonstrated that few-layer Sb nanosheets could work as a bifunctional catalyst in both HER and OER reactions, the two half-cell reactions of a water splitting reaction [19,20]. Using density functional theory, Kokabi et al. [21] found that the armchair edge of the antimonene nanosheet is more active than the zigzag edge. Recently, Lu et al. predicted that the activity of antimonene could be improved when transition metal atoms were doped into the lattice [22].

Antimonene flakes/nanosheets can be prepared by different methods, such as mechanical exfoliation [23], liquid exfoliation [9,17,18], vapor transport deposition [24], and molecular beam epitaxy (MBE) [15,25,26] as listed in Table 1. Usually, the flakes from mechanical exfoliation are of a high quality, but the yield is extremely low with small lateral sized flakes (~1 µm) and a poor and uncontrollable thickness uniformity. The liquid exfoliation usually provides a higher yield, but like mechanical exfoliation, the flakes are of small size with poor thickness uniformity. In addition, surfactant is usually required for the flakes to be suspended in the solution. Vapor transport deposition is used to deposit Sb flakes; however, it is still challenging to control the thickness using this technique. MBE is another method used to produce Sb flakes, especially monolayers of a very high quality. However, the domain sizes are typically restricted to only several tens of nanometers, and, usually, specific substrates are required for the epitaxy. Moreover, the process is not compatible with mass production. The controllable growth of large-scale continuous antimonene nanofilms with high uniformity is in high demand. 

Magnetron sputtering deposition is a classical method for thin film deposition and is widely used in industry. However, to date, no deposition of Sb nanofilms with magnetron sputtering has been demonstrated [27]. In this study, we demonstrated the deposition of Sb nanofilms with a low-power magnetron sputtering system. The deposited films had lateral dimensions of centimeters, and the thickness of the films could be easily controlled with sub-nm accuracy, which was confirmed with atomic force microscopy (AFM). Raman spectroscopy and transmission electron microscopy (TEM) verified that the deposited films were a polycrystalline β phase when deposited at high temperature, but the films had an amorphous nature when deposited at room temperature. The Sb nanofilms showed activity in the HER and OER reactions, and the films deposited at a high temperature showed better performance. 

## 2. Materials and Methods

Sputtering deposition: In this work, the Sb films were deposited with a low-power magnetron sputtering system. A piece of two-inch Sb target (purity: 99.995%) was used as a source, and SiO_2_/Si substrates were loaded as deposition substrates. For the deposition, the sputtering chamber was pumped down to 1 × 10^−6^ torr to remove oxygen and humidity; then, 30 sccm Ar gas was supplied to the chamber, and the pressure was adjusted to 3 × 10^−3^ torr for the sputtering deposition. The sputtering power was set to 1 W, and the deposition duration varied from 5 min to 20 min. The deposition was conducted at either room temperature (25 °C) or high temperature (300 °C). For the electrochemical tests, conductive carbon cloth was used as the substrate. 

Raman characterization: The Raman spectra of the samples were collected with a WITec 300R system (WITec, Ulm, Germany). The wavelength of the excitation laser was 532 nm, and the laser power intensity was less than 0.5 mW to avoid heating effect. 

TEM characterization: For TEM sample preparation, a layer of polyvinyl alcohol (PVA) film was spin-coated onto a SiO_2_/Si substrate before the Sb nanofilms were deposited. To prevent the PVA film from melting, the deposition temperature was set to a maximum of 170 °C for the high-temperature sample. After deposition, a small piece of the as-deposited sample was immersed in a deionized (DI) water droplet. After the PVA film was dissolved in the water droplet, the Sb nanofilm floated on the surface and was scooped up with a TEM grid. The grid was further rinsed in a large amount of DI water to remove the PVA residual. After they were dried in a vacuum box, the TEM samples were loaded into a JEM-2100F electron microscope (JEOL, Tokyo, Japan) for characterization. The acceleration voltage of the electron beam used was 200 KeV. 

Hydrogen evolution reaction (HER), oxygen evolution reaction (OER), and the electrochemical surface area (ECSA) were measured in a 1 M KOH solution using the carbon cloth as the substrate. The OER was conducted with cyclic voltammetry (CV) (Gamry Reference 600 by Gamry Instruments, Warminster, PA, USA) at a scan rate of 5 mV/s. The HER was conducted using linear sweep voltammetry (LSV) (Gamry Reference 600 by Gamry Instruments, USA) at the same scan rate. The ECSA test was performed under different scan rates, from 5 mV/s to 100 mV/s. Before immersion in the electrolyte, samples were covered with silicone paste to avoid solution entry beyond a reaction region with an area of 1 cm^2^.

## 3. Results and Discussions

**Figure 1:**
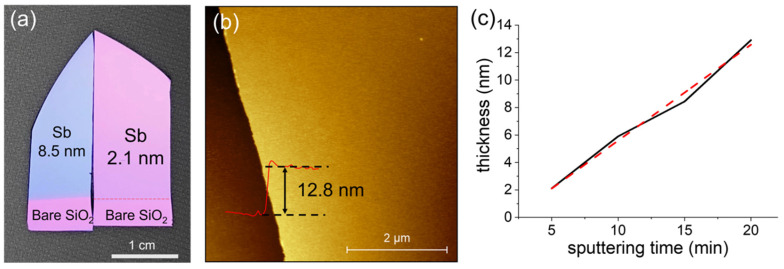
(**a**) Optical image of the Sb nanofilms deposited on SiO_2_/Si substrates for different durations (left piece: 15 min; right piece: 5 min) at room temperature. (**b**) Atomic force microscopic characterization of the as-deposited Sb nanofilm deposited for 20 min on a SiO_2_/Si substrate. (**c**) Thickness dependence on the deposition duration.

There are several methods to prepare antimonene nanoflakes; however, it is still challenging to prepare Sb films with a large lateral size and good thickness control [24,26,28]. In this work, the Sb nanofilms were deposited using a magnetron sputtering system with a sputtering power as low as 1 W at a pressure of 3 × 10^−3^ torr for different durations (5 min, 10 min, 15 min, and 20 min). After a deposition of 5 min, the film still looked like the color of the SiO_2_ (270 nm)/Si substrate, which meant that the film was quite thin. As the deposition duration increased, the film became gradually blue in color, as shown in Figure 1a for a sample deposited for 15 min. The thickness of each sample was confirmed using an atomic force microscope (AFM) (Dimension Edge by Bruker Corporation, Billerica, MA, USA). Figure 1b shows a topography image of a sample deposited for 20 min on a SiO_2_/Si substrate. The thickness of the film is 12.8 nm, and the surface roughness is as low as 609.1 pm over 1 µm^2^. The thickness depends on the sputtering duration linearly (Figure 1c) with an average deposition rate of 0.7 nm/min, which makes it easy to control the thickness of the films by changing the sputtering duration. 

**Figure 2:**
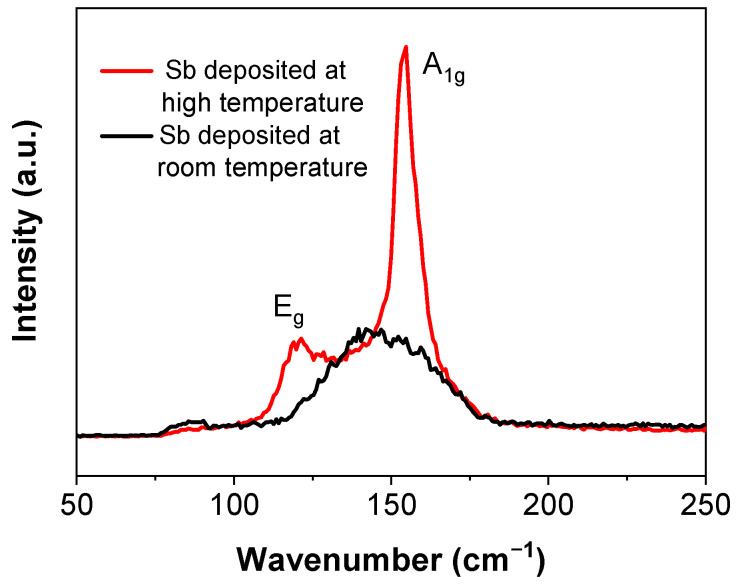
Raman Spectra of Sb films deposited at different temperatures.

Raman spectroscopy is sensitive to lattice vibrations and is an effective method to confirm the structure of the antimonene flakes. From the Sb nanofilm deposited at room temperature, only one broad peak appears at approximately 145 cm^−1^, with a full width at half peak of 42 cm^−1^ (Figure 2), which is distinct from the two peaks of the reported results [12,17]. This indicates that the film had a poor crystalline structure. If deposited at room temperature, it may be difficult for the sputtered atoms or small clusters to obtain enough energy to diffuse to form a crystalline structure on the substrate [29]. To improve the quality of the deposited film, the substrate was heated up to 300 °C in situ for the deposition. In contrast to the broad peak of the sample deposited at room temperature, the Raman spectrum of this sample shows two sharp peaks at 120 cm^−1^ and 154 cm^−1^, corresponding to the in-plane E_g_ vibration and the out-of-plane A_1g_ vibration, respectively. This is consistent to the reported results [19,24]. These two peaks indicate that the film is β phase and the crystalline structure was improved by depositing the film at a high temperature. 

**Figure 3:**
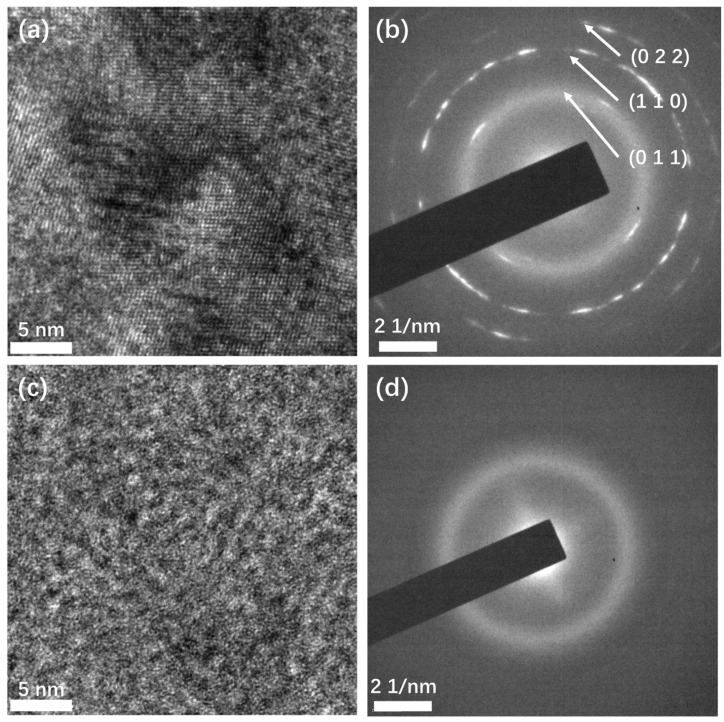
TEM characterization of the Sb films deposited at different temperatures. (**a**) HRTEM image and (**b**) SAED image of Sb film deposited at high temperature; (**c**) HRTEM and (**d**) SAED image of Sb film deposited at room temperature.

To confirm the crystalline structure, transmission electron microscopy was performed (Figure 3). Crystalline structures can be observed in the high-resolution TEM (HRTEM) image (Figure 3a) of the Sb films deposited at high temperatures. The different orientations of the crystal grains indicate that the film is polycrystalline. This structure was also confirmed by selected area electron diffraction (SAED). In the SAED image (Figure 3b), the diffracted electrons formed a series of discontinuous circles with short arcs, instead of points, indicating the film is polycrystalline, which is in agreement with the HRTEM result. From the center out, the corresponding interplane distances of the first three circles were calculated to be 0.359 nm, 0.218 nm, and 0.178 nm, which match the interplane distances of the crystal planes (011), (110), and (022) of β phase antimony, respectively. This confirms that the films deposited at high temperature are β phase, consistent with the Raman test result. In contrast, in the HRTEM image (Figure 3c) of the Sb film deposited at room temperature, no crystalline domain is observed, and no diffraction spots or circles appear in the SAED image (Figure 3d). This confirms that the film had an amorphous structure. The TEM characterization result is consistent with the result of Raman spectroscopy. 

Sb flakes are potential catalysts for the water splitting reaction, in which the Sb flakes can act as catalysts for both the oxygen evolution reaction and hydrogen evolution reaction [19,21,22]. In this work, the electrochemical activity of the deposited Sb films was evaluated. The effect of the deposition temperature on the catalyst activity was also studied by depositing Sb films at both room temperature and high temperature (300 °C). The OER, HER, and electrochemical active surface area (ECSA) were measured in 1 M KOH solution using carbon cloth as substrate.

**Figure 4:**
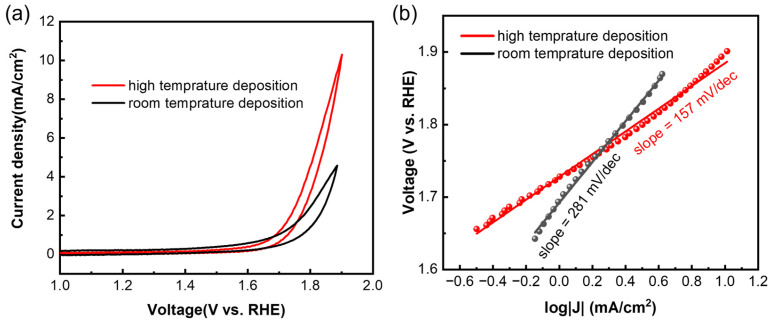
(**a**) Cyclic voltammetry test of Sb nanofilms deposited at different temperatures, and (**b**) corresponding Tafel slopes.

For the OER, the samples were tested using cyclic voltammetry (CV) at a scan rate of 5 mV/s. Both the films deposited at high temperature and room temperature showed OER activity (Figure 4a), and the high-temperature sample showed a higher current density at the same voltage. Overpotential, also known as onset potential, is a key parameter to evaluate the activity of a catalyst, and it means the excess potential that is required to overcome the kinetic barrier [19,20]. At a current density of 1 mA/cm^2^, the overpotential for both samples was calculated to be 1.6 V vs. reversible hydrogen electrode (RHE), which is similar to the values of the exfoliated samples [19]. The Tafel slope is another key parameter, and a smaller Tafel slope means faster electrocatalytic reaction kinetics [20]. The Tafel slope (Figure 4b) of the high-temperature sample (157 mV/dec) is lower than that of the room-temperature sample (281 mV/dec), which means the high-temperature sample had faster OER kinetics. The Tafel slope of the sample deposited at high temperature is also lower than that of the samples from the liquid exfoliation process [19], indicating a better quality of the samples prepared using low-power magnetron sputtering deposition. 

**Figure 5:**
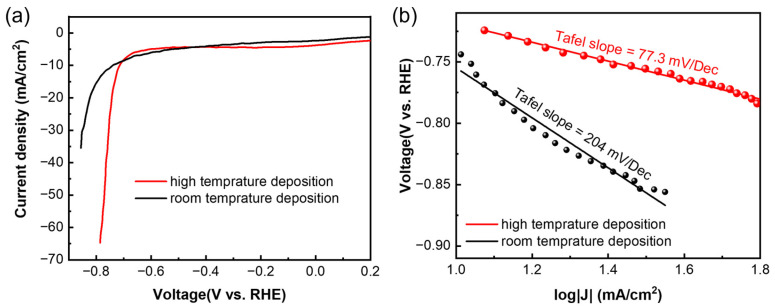
(**a**) Linear sweep voltammetry test of Sb nanofilms deposited at different temperatures, and (**b**) corresponding Tafel slopes.

As for the HER, it was tested using linear sweep voltammetry (LSV) at a scan rate of 5 mV/s. Similar to the OER, both the films showed HER activity (Figure 5a), and the higher-temperature sample showed higher current density at the same voltage. The overpotentials were calculated to be −0.64 V vs. RHE and −0.61 V vs. RHE for the samples deposited at high temperature and room temperature, respectively, which is similar to the other reported values [19,30]. The Tafel slope (Figure 5b) of the high-temperature sample (77.3 mV/dec) is much lower than that of the room sample (204 mV/dec), which indicates faster HER kinetics of the high-temperature sample. It is also lower than the values reported by Ren et al. [19], indicating a better quality in the deposited film.

**Figure 6:**
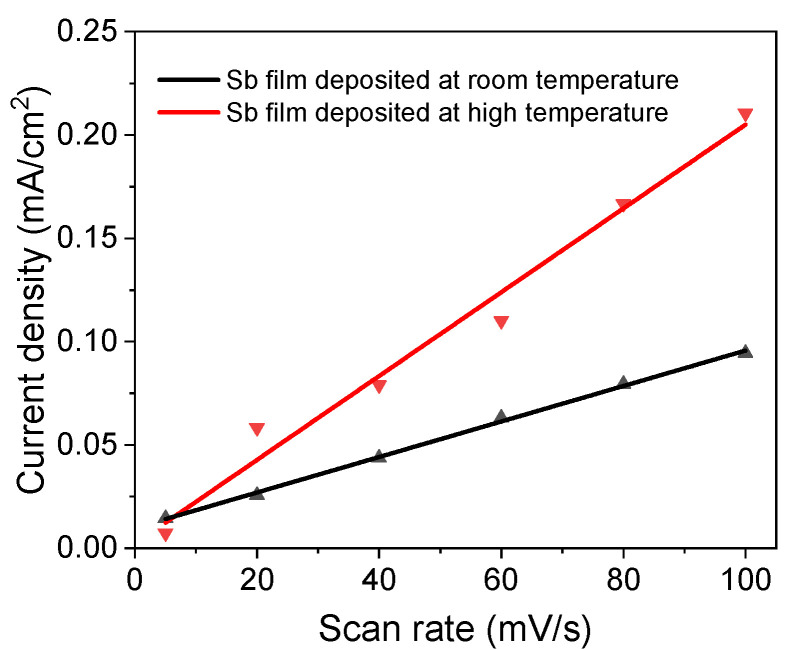
Electrochemical active surface area test of Sb nanofilms deposited at different temperatures.

An ECSA test was performed under different scan rates from 5 mV/s to 100 mV/s. The slopes of the current density vs. scan rate (Figure 6) are 2.03 µF/cm^2^ and 0.86 µF/cm^2^ for the high-temperature sample and room-temperature sample, respectively. Therefore, the ECSA ratio of the sample deposited at high temperature to the sample deposited at room-temperature is 2.4, which means that the sample deposited at high temperature is more active than the room-temperature sample, since the film thickness for both samples is the same. 

## 4. Conclusions

In this work, the deposition of antimonene nanofilms was performed with a low-power magnetron sputtering system. With this method, centimeter-sized Sb nanofilms were deposited with controllable thickness and low roughness, with a deposition rate of 0.7 nm/min. The crystalline structure of the nanofilms was determined by the deposition temperature. When deposited at room temperature, the films had an amorphous structure, while when they were deposited at high temperature, the films had a polycrystalline structure, which was confirmed by both Raman spectroscopy and TEM characterization. The electrochemical activity of the films was also evaluated using OER and HER tests. The films deposited at high temperature showed higher activity than those deposited at room temperature. The better performance of the films deposited at high temperature may be attributed to the highly crystalline structure compared to the amorphous structure of the Sb films deposited at room temperature.

## Figures and Tables

**Table 1:** Comparison between different preparation methods for antimonene nanofilms.

Preparation Methods	Advantages	Disadvantages	Refs
Mechanical exfoliation	Simple process High quality	Low yield Small flake size (~1 µm) Poor uniformity	[23]
Liquid exfoliation	Simple process High yield	Small size (<1 µm) Poor uniformity Surfactant required	[9,17,18,24]
Vapor transport deposition	Simple process Suitable to films	Thick flakes Poor uniformity of thickness	[24]
MBE	High quality Monolayers High uniformity	Small size (tens of nm) Specific substrates required Expensive complicated setup	[15,25,26]
Low-power sputtering deposition	Controllable thickness (sub-nm accuracy) Large size films (centimeter scale) Applicable to various substrates	Small domain size	This work
